# Polyvinylpyrrolidone-Modified Taxifolin Liposomes Promote Liver Repair by Modulating Autophagy to Inhibit Activation of the TLR4/NF-κB Signaling Pathway

**DOI:** 10.3389/fbioe.2022.860515

**Published:** 2022-06-01

**Authors:** Qiteng Ding, Wencong Liu, Xinglong Liu, Chuanbo Ding, Yingchun Zhao, Ling Dong, Huiying Chen, Shuwen Sun, Yiwen Zhang, Jinping Zhang, Ming Wu

**Affiliations:** ^1^ College of Chinese Medicinal Materials, Jilin Agricultural University, Jilin, China; ^2^ College of Life Science, Jilin Agricultural University, Jilin, China

**Keywords:** polyvinylpyrrolidone-K30, taxifolin, liposomes, acute liver injury, TLR4/NF-κB, autophagy

## Abstract

Taxifolin (TAX) is a hepatoprotective flavanol compound, which is severely limited by poor solubility and low bioavailability. Liposomes (Lips) are used as well-recognized drug carrier systems that improve the water solubility and bioavailability of drugs, but are easily damaged by gastric juice after oral administration, resulting in the release of drugs in the gastric juice. Therefore, it is important to find materials that modify liposomes and avoid the destruction of the liposomal phospholipid bilayer structure by the gastrointestinal environment. Taxifolin liposomes (TAX-Lips) were modified by polyvinylpyrrolidone-k30 (PVP-TAX-Lips) and manufactured using a thin-film hydration technique. Particle size (109.27 ± 0.50 nm), zeta potential (−51.12 ± 3.79 mV), polydispersity coefficient (PDI) (0.189 ± 0.007), and EE (84.7 ± 0.2%) of PVP-TAX-Lips were studied. In addition, the results of *in vitro* release experiments indicated that the cumulative release rates of TAX-Lips and PVP-TAX-Lips were 89.73 ± 5.18% and 65.66 ± 4.86% in the simulated gastric fluid after 24 h, respectively, while the cumulative release rates were 68.20 ± 4.98% and 55.66 ± 3.92% in the simulated intestinal fluid after 24 h, respectively. Moreover, PVP-TAX-Lips were able to reverse lipopolysaccharide and D-galactosamine (LPS/D-GalN)-induced acute liver injury (ALI) by inducing autophagy to inhibit the expression levels of the TLR4/NF-κB signaling pathway and inflammatory factors, which suggested that PVP-TAX-Lips played an important role in the prevention of ALI and also provided a promising drug delivery system for the application of TAX.

## 1 Introduction

The liver plays an important role in human metabolism, immune response, protein synthesis, and removal of pathogens and toxins (Ma et al., 2014). Acute liver injury (ALI) is an inflammatory disease induced by many factors including drugs such as acetaminophen (APAP), carbon tetrachloride (CCl4), and lipopolysaccharide and D-galactosamine (LPS/D-GalN) (Bohm et al., 2016; Khoury et al., 2017; Woolbright and Jaeschke 2017; Liu Wei et al., 2018; Huang et al., 2021). Regardless of its etiology, ALI can induce liver fibrosis, cirrhosis, and even liver cancer, which is the 12th most common malignancy in the United States (Koyama and Brenner 2017). In addition, previous studies have shown that ALI occurs by complex mechanisms related to inflammation, oxidation, and autophagy (Liu Wei et al., 2018; Liu et al., 2020; Miao et al., 2020). LPS is a typical toxic component of Gram-negative bacteria, and the toxicity of LPS can be increased by D-GalN. Hence, the clinical ALI in mice will be induced by LPS/D-GalN within a few hours (Li et al., 2020), a model commonly used to study and develop new hepatoprotective drugs, such as Taxifolin (TAX), which is a natural product with excellent hepatoprotective effects (Liu Aiyun et al., 2018; Zhou Honghong et al., 2018; Yang et al., 2019).

TAX [(2R, 3R) 3, 3ʹ, 4ʹ, 5, 7-pentahydroxy flavan-4-one], also known as dihydroquercetin, is a flavanol compound found in conifers such as Pinus Roxburghe and Siberian larch as well as in plant-based foods such as vegetables, fruits, wine, tea, and cocoa. Interestingly, numerous studies have shown that TAX has anti-inflammatory, antioxidant, antidiabetic, and hepatoprotective effects (Sun et al., 2014; Cai et al., 2018; Lektemur Alpan et al., 2020; Ahiskali et al., 2021). TAX has been approved as a novel food ingredient by many countries including the US, United Kingdom, European Commission, and China (Ding et al., 2021). However, poor water solubility and oral utilization of TAX limit its application in the medical industry (Shikov et al., 2009). Therefore, many studies have reported methods to increase the solubility of TAX. For example, TAX was prepared as a powder by a supercritical antisolvent to improve the solubility of TAX in water (Zu et al., 2012). TAX has also been prepared as liposomes for use in beverage and skin preparation (Kim et al., 2019; Hasibi et al., 2019), but the mechanism of TAX-Lips *in vivo* have not been explored in depth. Liposomes are recognized as drug carriers with a phospholipid bilayer structure which are biocompatible and capable of improving drug absorption, reducing drug toxicity, and increasing bioavailability of unstable or insoluble drugs (Ong et al., 2016; Pan et al., 2018; Yuan et al., 2017). Hence, the encapsulation of TAX in liposomes is an effective way to increase the bioavailability of TAX.

Unfortunately, current applications of liposomes are still limited to intravenous injection therapy, as oral treatment of liposomes faces great challenges, such as the damage in the gastrointestinal tract and the poor permeability of the gastrointestinal epithelium (He et al., 2019; Wang et al., 2017; Zhou et al, 2020). Consequently, numerous strategies have been developed to avoid the destruction of liposomes by the gastrointestinal tract in order to improve the bioavailability of their cargoes, such as modifications with polyethylene glycol (PEG), maltodextrin, and chitosan (Gurturk et al., 2017; Ota et al., 2018; Stenger Moura et al., 2019; Steffes et al., 2020). Polyvinylpyrrolidone (PVP) has been reported to form a furry protective layer on the outer layer of liposomes that may avoid drug leakage caused by the complex gastrointestinal environment (Liu et al., 2017b). Therefore, we explored the preparation, characterization, and hepatoprotective activity of TAX-Lips modified by PVP (PVP-TAX-Lips) to develop a new dosage form, able to increase the bioavailability of TAX. The current research on liposomes is usually focused on *in vivo* pharmacokinetics and *in vitro* cytotoxicity, but *in vivo* mechanisms of action are sometimes still unclear (Grillone et al., 2017; Zhou Chuchu et al., 2018; Wang et al., 2019; Yao et al., 2019; Zhang et al., 2019; Jagwani et al., 2020; Le et al., 2021). Therefore, this study was conducted to evaluate the hepatoprotective effect of PVP-TAX-Lips and its mechanism in LPS/D-GalN-induced ALI.

## 2 Materials and Methods

### 2.1 Reagents and Materials

Taxifolin was purchased from the National Institutes for Food and Drug Control of China, batch No: 111816–201102, with a purity of 98.0%. Soy phosphatidylcholine (SPC) and Cholesterol (Chol) were purchased from Shanghai Macklin Biochemical Co., Ltd. (Shanghai, China). Polyvinylpyrrolidone-K30 (PVP-K30) was obtained from Anhui Shanhe Pharmaceutical Excipients Co., Ltd. (Huaihe, China). Chloromethane and methanol were provided by Shanghai Sinopharm Chemical Reagent Co., Ltd. (Shanghai, China). Phosphate buffer saline (PBS) powder was purchased from Solarbio Biotechnology Co., Ltd. (Beijing, China), and the water used in the experiment was distilled water. Lipopolysaccharide (LPS) from *Escherichia coli* 055: B5 was obtained from Sigma-Aldrich Co., Ltd. (Shanghai, China). D-galactosamine (D-GalN) was acquired from Aladdin Reagent Database Co., Ltd. (Shanghai, China). The commercial assay kit of alanine aminotransferase (ALT), aspartate aminotransferase (AST), glutathione (GSH), superoxide dismutase (SOD), malondialdehyde (MDA), and hematoxylin-eosin (H&E) were purchased from Nanjing Jiancheng Bioengineering Research Institute Co., Ltd. (Nanjing, China). Rabbit primary polyclonal antibodies to Toll-like receptor 4 (TLR4), myeloid differentiation factor88 (MyD88), nuclear factor-kappa B (NF-κB, P65), sequestosome-1 (P62), light chain 3 (LC3), mouse Interleukin-1β (IL-1β), inducible nitric oxide synthase (iNOS), tumor necrosis factor α (TNF-α), β-actin, Autophagy related 7 (ATG7), Autophagy related 5 (ATG5), inhibitor kappa B alpha (IκBα), phospho-inhibitor kappa B alpha (p-IκBα), goat anti-rabbit IgG horseradish peroxidase (HRP)-conjugated secondary antibody, and goat anti-mouse IgG-HRP-conjugated secondary antibody were bought from Proteintech (Wuhan, China).

### 2.2 Preparation of TAX-Lips and PVP-TAX-Lips

**FIGURE 1:**
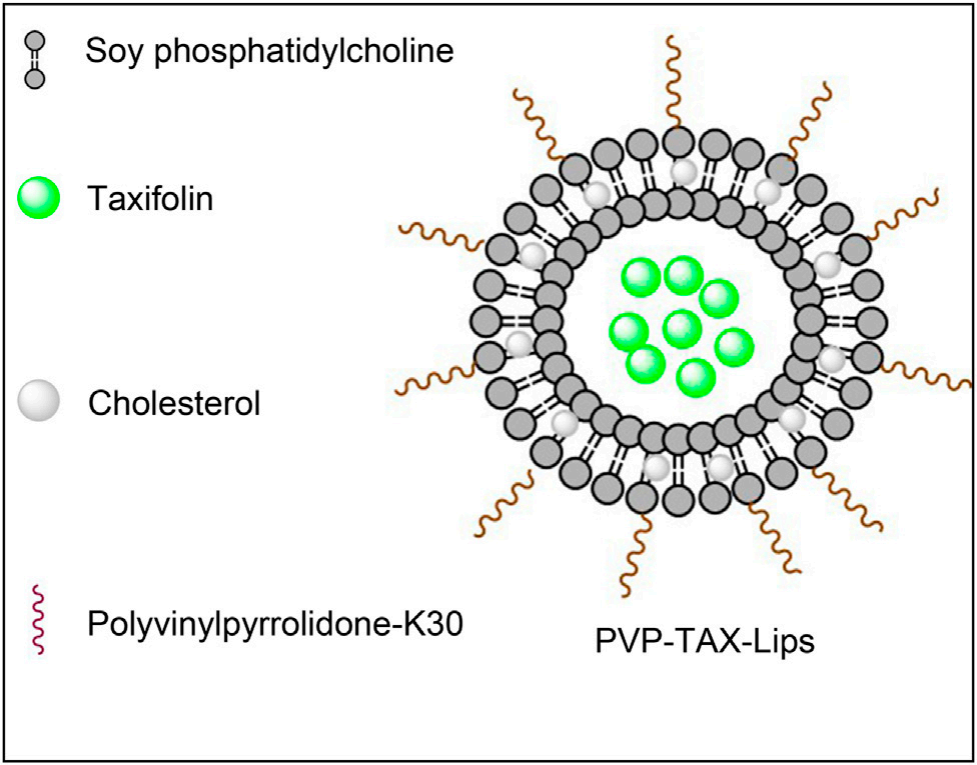
The schematic diagram of the structure of PVP-TAX-Lips.

TAX-Lips and PVP-TAX-Lips were prepared by a thin-film hydration technique. To briefly describe the preparation process, TAX (10 mg), SPC (60 mg), and Chol (10 mg) were weighed and dissolved in chloroform and methanol (4:1). Thereafter, chloroform and methanol were removed in a rotary evaporator under vacuum at 35°C to obtain a thin film, and then 10 ml of PBS aqueous solution containing 1% PVP-K30 was added to the hydrate to obtain the liposomes. Next, the solution was ultrasonicated at 100 W for 5 min in an ice bath to obtain the PVP-TAX-Lips. The schematic structure of PVP-TAX-Lips is shown in Figure 1. On the other hand, TAX-Lips were prepared following the same procedure but without the addition of PVP-K30 in the abovementioned steps.

### 2.3 Characterization of TAX-Lips and PVP-TAX-Lips

#### 2.3.1 Encapsulation Efficiency

Due to the poor water solubility of TAX, the nonencapsulated or free drug can be removed by centrifugation (Han et al., 2020). For that, 0.5 ml of TAX-Lips or PVP-TAX-Lips solution was centrifuged at 12000 rpm at 4°C for 30 min. Then, the amount of encapsulated TAX (W_1_) in the liposomes was obtained by the addition of 10 ml of methanol to the supernatant followed by ultrasonic breaking for 5 min, while the total TAX amount (W_total_) in the liposome solution was obtained by taking the same volume of TAX-Lips or PVP-TAX-Lips solution and then adding 10 ml of methanol for ultrasonic crushing for 5 min. TAX was quantified by high performance liquid chromatography (HPLC) (Waters, e2695, United States) with UV detection at 290 nm. Methanol (45%) and water (55%) were used as mobile phase, at 1.0 ml/min, and a COSMOSIL C18-PAQ column as stationary phase (4.6 × 250 mm, 5 μm), The encapsulation efficiency (EE) was calculated by the following equation:

$$EE\left(\%\right)=\frac{W_{1}}{W_{total}}\times 100\%.$$

#### 2.3.2 Particle Size and Zeta Potential Analysis of TAX-Lips and PVP-TAX-Lips

Nanobrook 90 plus zeta (Brookhaven, United States) was used to analyze the particle size, zeta potential, and polydispersity coefficient (PDI) of TAX-Lips and PVP-TAX-Lips. The measurement temperature was maintained at 25°C, while the scattering angle was set to 90° C. The sample was diluted 10 times with double distilled water before the test. All tests were performed 3 times in parallel.

#### 2.3.3 Transmission Electron Microscopy

The morphology of PVP-TAX-Lips and TAX-Lips was observed by transmission electron microscopy (TEM, JEM-1200 EX, Hitachi, Japan). The prepared liposomes were diluted 10 times with double distilled water. TAX-Lips and PVP-TAX-Lips were supported on a copper grid and negatively stained with 0.2% (w/v) phosphotungstic acid.

#### 2.3.4 Fourier Transform Infrared Spectroscopy

All the samples were lyophilized for at least 48 h by using a freeze-drier (Alpha 1-2 LD plus; BMH Instrument Co., Ltd., Beijing, China) before the measurements. The dried samples and KBr were grounded into homogeneous powder in a mortar and pressed into suitable tablets with appropriate thickness. The infrared characteristic peaks of pure TAX, TAX-Lips, PVP-TAX-Lips, and the physical mixture of SPC, TAX, and PVP-K30 were measured in the range of 400–4,000 cm^−1^, using a Fourier transform infrared spectrophotometer (FTIR) (CT Norwalk PerkinElmer, United States).

### 2.4 *In Vitro* Drug Release

The cumulative *in vitro* release rates of PVP-TAX-Lips, TAX-Lips, and free TAX over 24 h were determined by dialysis. Two mL of PVP-TAX-Lips, TAX-Lips, and free TAX were added to the dialysis bag and then placed in 200 ml of two release media: simulated gastric (pH = 1.2) and simulated intestinal fluid (pH = 6.8). The samples were shaken at 100 rpm in a bath at 37°C, and 2 ml aliquots of release medium were taken at 0, 0.5, 1, 2, 4, 6, 8, 10, 12, and 24 h. The aliquots withdrawn were replaced with the same volume of fresh medium at 37°C. TAX was quantified by HPLC (Waters, e2695, United States) with UV detection at 290 nm. Methanol (45%) and water (55%) were used as mobile phase at 1.0 ml/min, and a COSMOSIL C18-PAQ column as stationary phase (4.6 × 250 mm, 5 μm). The cumulative release of TAX from free TAX, TAX-Lips, and PVP-TAX-Lips at different time points were calculated according to the following equation:

$$\mathrm{Cumulative release rate}\left(\%\right)=\left[V_{1}\times \;\left(C_{1}+C_{2}+\cdots +\;C_{i-1}\right)+V_{2}\times C_{i}\right]/\left(V_{0}\times C_{0}\right)\;\times 100\%.$$

In the equation, V_1_: sampling volume at each time point; V_2_: volume of dissolution medium; C_1_∼C_i_: concentration of substance measured at each time point; V_0_: volume of the medium at 100% dissolution; and C_0_: concentration of a substance at 100% dissolution.

### 2.5 *In Vivo* Experiment

#### 2.5.1 Animals and Experimental Plan

40 male ICR mice (6–8 weeks old; weight 23 ± 2 g) were purchased from Changchun YISI Experimental Animal Co., Ltd. (Certificate of Quality: No. SCXK (JI) -2019–0,006, Changchun, China). The animals were given adequate food and water, and kept in a suitable environment (24 ± 2°C, 55 ± 10% humidity, 12 h light-dark cycle). The experiments were conducted by the National Institutes of Health Guide for Laboratory Animals Care and Use and the Laboratory Animal Management Committee of Jilin Agricultural University, and approved by the Animal Investigational Morals Committee of Jilin Agricultural University, with the ethics approval number 2019–08–28–002.

The mice were randomly divided into four groups after 1 week of adaptive feeding (*n* = 10 in each group). The control group and the model group were given physiological saline for 14 days, and the TAX-Lips group and the PVP-TAX-Lips group were given the same dose of TAX (50 mg/kg) for 14 days by intragastric administration. The mice were induced by intraperitoneal injection of LPS (10 μg/kg) and D-galactose (700 mg/kg) for ALI in all the groups except the control group, 1 h after the administration on the last day, and then anesthetized by intraperitoneal injection of 70 mg/kg pentobarbital sodium after 6 h (Shanghai Beizhuo Biochemical and Technological Co., Ltd., Shanghai, China). The serum was collected by centrifugation at 3,500 ×g at 4°C for 10 min. Then, the anaesthetized mice were sacrificed by dislocation and the liver tissues were immediately dissected, washed with cold saline, blotted on a filter paper, and the shape and size of the tissues were observed. The liver tissues were taken partially and preserved in 10% formaldehyde, while the remaining liver and serum were stored at −80°C. The liver index was calculated using the following equation:

Liver index(%)=liver weight(g)/final weight of the mice(g)×100%.

### 2.6 Histological Analysis

The liver tissues were fixed in 10% formalin solution and embedded in paraffin, and then cut into 5 μm thickness slices. The pathological sections were examined to assess the extent of ALI with hematoxylin and eosin (H&E) staining kits for further observation under a light microscope (Bio-Rad, Hercules, United States) (100× and 200×). The degree of liver necrosis was assessed and quantitatively scored by necrotic area, fat vacuolation, and inflammatory cell infiltration (Liu Aiyun et al., 2018; Yan et al., 2018).

### 2.7 Biochemical Analysis

The ALT and AST levels in the serum were quantified according to the manufacturer’s protocol using the clinical autoanalyzer (Hitachi, Japan) and a commercial kit (Roche Diagnostics, Mannheim, Germany). GSH, SOD, and MDA levels in the liver tissue were quantified according to the manufacturer’s protocol *via* commercial kits (Nanjing Jiancheng Bioengineering Research Institute Co., Ltd., Nanjing, China).

### 2.8 Western Blot Analysis

The expression levels of iNOS, TNF-α, IL-1β, TLR4, MyD88, IκBα, p-IκBα, P65, β-actin, LC3, P62, ATG7, and ATG5 were detected by western blotting. The liver tissues were lysed using RIPA lysis buffer and phosphatase inhibitor (Sangon Biotech Co., Ltd., Shanghai, China), and then 5x loading buffer solution was added and heated at 100°C for 10 min. The proteins were transferred to polyvinylidene fluoride (PVDF) membranes by 10% SDS-PAGE separation and blocked in 10% nonfat milk for 2 h at room temperature, and the membranes were incubated overnight at 4°C with primary antibodies of iNOS, TNF-α, IL-1β, TLR4, MyD88, IκBα, p-IκBα, P65, β-actin, LC3, P62, ATG7, and ATG5, and then further incubated at room temperature with secondary antibodies for 1 h. The protein band signals were detected with ECL luminescent solution (Pierce Chemical Co., Ltd. Rockford, IL, United States), and the intensity of bands was quantified using ImageJ software.

### 2.9 Statistical Analysis

Statistical analysis was performed using SPSS 19.0 software (Chicago, IL, United States). The data are presented as the mean ± standard deviation (mean ± SD). Statistical analysis was conducted by one-way analysis of variance (one-way ANOVA) with post hoc comparisons of the least significant difference (LSD). The variances between the groups were tested for significance, and the statistical significance was set at *p* < 0.05, *p* < 0.01, or *p* < 0.001. GraphPad Prism software package 8.2 was used for figures.

## 3 Results

### 3.1 Encapsulation Efficiency

**FIGURE 2:**
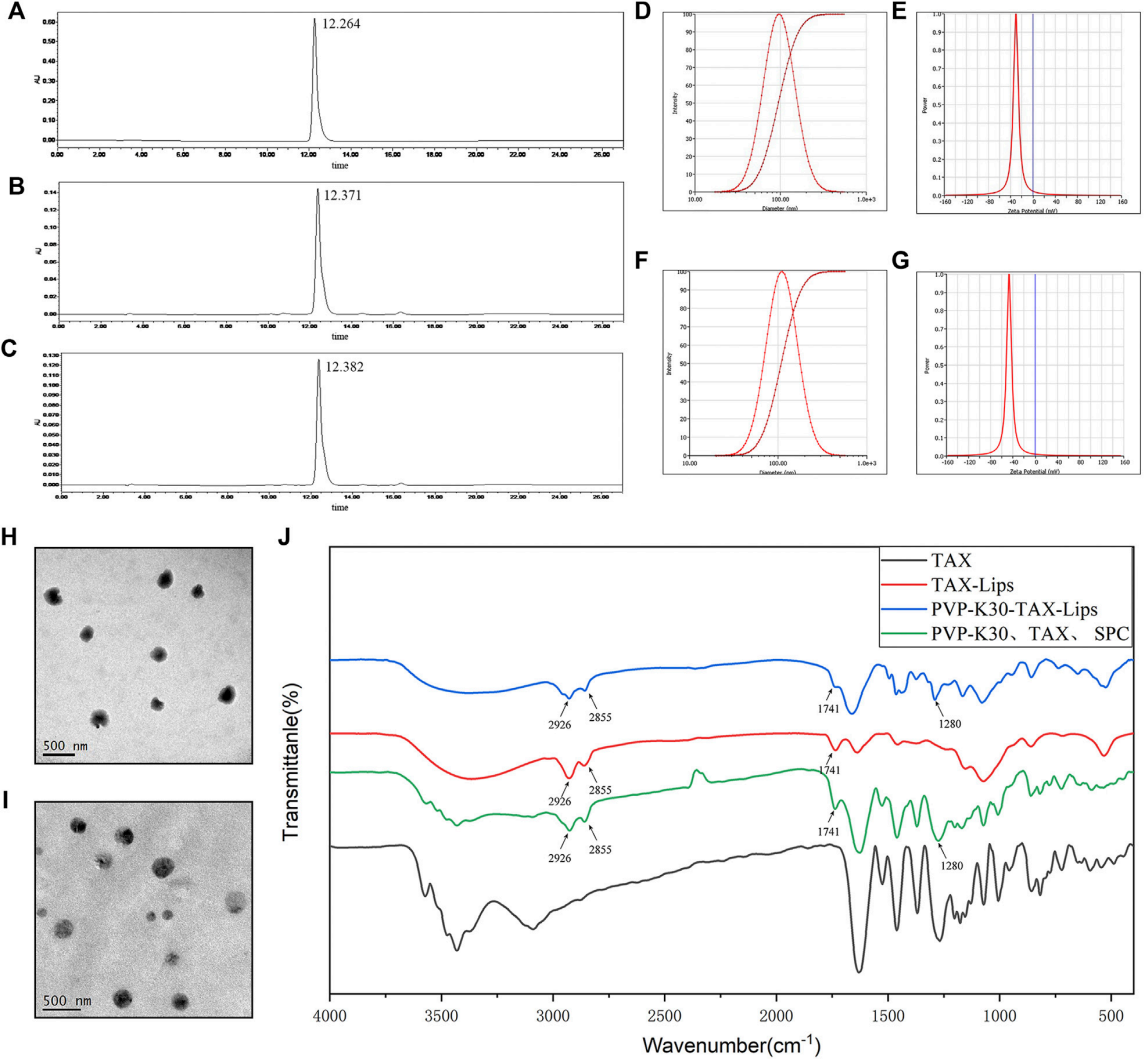
Characterization of TAX-Lips and PVP-TAX-Lips. **(A)** The HPLC chromatogram of TAX standard. **(B)** The HPLC chromatogram of TAX-Lips. **(C)** The HPLC chromatogram of PVP-TAX-Lips. **(D)** Particle size of TAX-Lips. **(E)** Zeta potential of TAX-Lips. **(F)** Particle size of PVP-TAX-Lips. **(G)** Zeta potential of PVP-TAX-Lips. **(H)** TEM image TAX-Lips. **(I)** TEM image of PVP-TAX-Lips. **(J)** FTIR spectra of TAX-Lips, PVP-TAX-Lips, and pure TAX, and a physical mixture of PVP-K30, TAX, and SPC.

The HPLC chromatograms of TAX (standard), TAX-Lips, and PVP-TAX-Lips are shown in Figures 2A–C. The EE of TAX-Lips was 91.3 ± 1.0% and that of PVP-TAX-Lips was 84.7 ± 0.2% (Table 1). The EE of PVP-TAX-Lips was slightly decreased compared to that of TAX-Lips, which may be due to the fact that PVP prevented a small portion of TAX from entering the liposome surface.

**TABLE 1:** Particle size, zeta potential, PDI, and EE of TAX-Lips and PVP-TAX-Lips.

Group	Particle size (nm)	PDI	Zeta potential (mV)	EE (%)
TAX-Lips	96.18 ± 0.66	0.186 ± 0.012	−33.75 ± 6.42	91.3 ± 1.0
PVP-TAX-Lips	109.27 ± 0.50	0.189 ± 0.007	−51.12 ± 3.79	84.7 ± 0.3

Data are presented as the mean ± SD (n = 3).

### 3.2 Measurement Results of the Particle Size, Polydispersity Coefficient, Zeta Potential, and Image of Liposomes by TEM

The results of particle size, PDI, and zeta potential are shown in Figures 2D–G. TAX-Lips showed a particle size of 96.18 ± 0.66 nm, PDI of 0.186 ± 0.012, and zeta potential of -33.75 ± 3.76 mV (Table 1; Figures 2D,E), while PVP-TAX-Lips showed a particle size of 109.27 ± 0.50 nm, PDI of 0.189 ± 0.007, and zeta potential of −51.12 ± 3.79 mV (Table 1; Figures 2F,G). The particle size and zeta potential of PVP-TAX-Lips were slightly increased compared with TAX-Lips, and TEM images showed that TAX-Lips and PVP-TAX-Lips were spherical structures with good dispersion, which proved that PVP modified TAX-Lips and did not affect the structure and dispersion of liposomes (Figures 2H,I).

### 3.3 Fourier Transform Infrared Spectroscopy Analysis

The FTIR characteristic peaks of pure TAX, PVP-TAX-Lips, and TAX-Lips, and the physical mixture of SPC, TAX, and PVP-K30 are shown in Figure 2J. The FTIR results revealed that the CH_2_ vibration of TAX-Lips and PVP-TAX-Lips at 2925 and 2854 cm^−1^ (Pan et al., 2018), respectively, the C=O symmetric stretching vibration at 1741 cm^−1^, and the C=N vibration at 1,280 cm^−1^ for PVP-TAX-Lips are the characteristic absorption peak of PVP (Jouyandeh et al., 2019). Interestingly, the characteristic peak of TAX in the range of 1,000–400 cm^−1^ disappears completely in TAX-Lips and PVP-TAX-Lips. Therefore, these experimental results suggest that PVP successfully modified TAX-Lips and formed an outer layer on the outside.

### 3.4 The Cumulative Rate of Drug Release *In Vitro*

**FIGURE 3:**
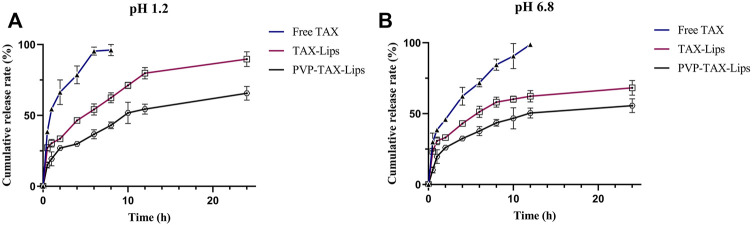
**(A)** *In vitro* release profiles of free TAX, TAX-Lips, and PVP-TAX-Lips in simulated gastric juice (pH = 1.2). **(B)** *In vitro* release profiles of free TAX, TAX-Lips, and PVP-TAX-Lips in simulated intestinal fluids juice (pH = 6.8) (mean ± SD, *n* = 3).

*In vitro* release profiles of TAX-Lips, PVP-TAX-Lips, and free TAX are shown in Figure 3. The results indicate that both the liposomes exhibited some slow-release properties in the simulated gastric fluid (pH = 1.2) and simulated intestinal fluid (pH = 6.8) compared to free TAX. The cumulative release rate of free TAX in the simulated gastric juice reached 95.40 ± 2.86% after 6 h, and was completely released after 8 h. The cumulative release rate of free TAX in the simulated intestinal fluid reached 90.60 ± 8.82% within 10 h and was completely released after 12 h. The rapid release of drug from TAX-Lips and PVP-TAX-Lips during 0–2 h was probably due to the rapid diffusion of unencapsulated drug and sudden release of liposomes, and the drug started to be released slowly after 2 h, reaching a maximum release of 89.73 ± 5.18% for TAX-Lips and 65.66 ± 4.86% for PVP-TAX-Lips after 24 h in the simulated gastric fluid. On the other hand, the cumulative release rate was 68.20 ± 4.98% for TAX-Lips and 55.66 ± 3.92% for PVP-TAX-Lips during 24 h in the simulated intestinal fluid.

The results of *in vitro* release experiments showed that PVP-TAX-Lips had a slow-release effect in the simulated gastrointestinal fluids.

### 3.5 The Role of PVP-TAX-Lips in Protecting the Liver

#### 3.5.1 PVP-TAX-Lips Improves LPS/D-GalN Induced Liver Morphological Abnormalities in Mice

**FIGURE 4:**
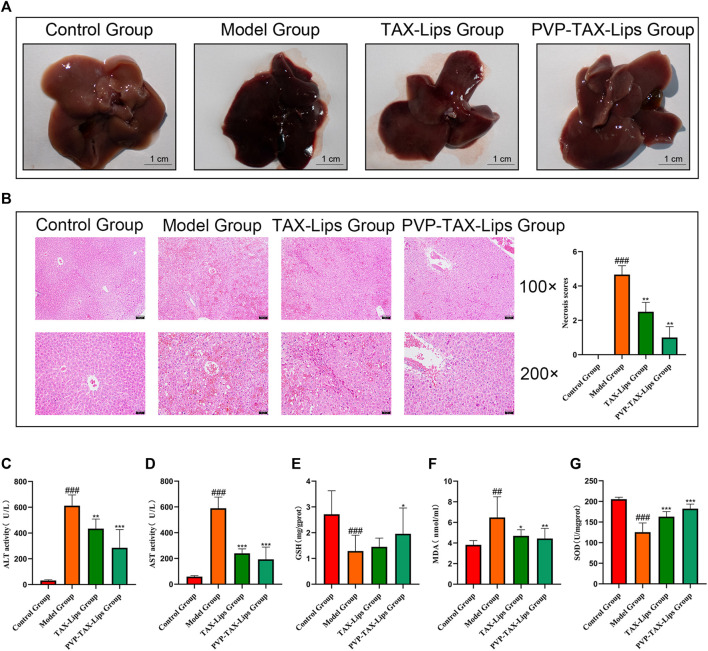
Acute liver injury in mice. **(A)** Effects of TAX-Lips and PVP-TAX-Lips on the appearance of the liver in mice. **(B)** H&E-stained liver tissue sections, with magnifications of ×100 and 200X, and the necrosis score of the liver. **(C,D)** ALT and AST levels in mice serum. **(E–G)** GSH, MDA, and SOD content in the mice liver tissue. As compared with the control group, ##*p <* 0.01,###*p <* 0.001; As compared with the model group, *p < 0.05, **p < 0.01, ***p < 0.001. The data are presented as the mean ± SD (n = 8).

The morphological appearance of the liver of mice is presented in Figure 4A. After 6 h, the liver of the model group showed obvious hemorrhagic damage due to the intraperitoneal injection of LPS/D-GalN, while the liver appearance of the mice treated with TAX-Lips and PVP-TAX-Lips was significantly improved. The results of H&E staining histopathology and the necrosis score are shown in Figure 4B. The liver tissue of the control group showed no abnormalities and structural integrity, with the hepatocytes arranged radially along the central vein with dense interstitial spaces. Conversely, the model group had disturbed hepatocyte arrangement, severely damaged the liver structure, increased fat vacuoles, and obvious inflammatory cell infiltration. PVP-TAX-Lips significantly reversed LPS/D-GalN-induced ALI without significant hepatocyte damage, according to the degree of hepatocyte necrosis represented by quantitative scoring of the histopathological results. It is worth mentioning that hepatocyte necrosis was significantly improved by PVP-TAX-Lips treatment compared to the model group (*p* < 0.01). The scores ranged from 0 to 5, with scores of 1-3 for mild liver injury, 3-4 for moderate liver injury, and 4-5 for severe liver injury.

#### 3.5.2 PVP-TAX-Lips Reversed the Increase of Liver Index in Mice With ALI Induced by LPS/D-GalN

The results in Table 2 showed that TAX-Lips and PVP-TAX-Lips had no effect on the body weight of mice after 14 days of treatment. The liver weight of mice in the model group was significantly increased compared with the control group (*p* < 0.001; Table 2), but the liver weight of mice treated with TAX-Lips and PVP-TAX-Lips was significantly reduced compared with the model group (*p* < 0.01; Table 2). The liver index was significantly increased in the model group (7.5 ± 1.3%) compared with the control group (*p* < 0.01; Table 2), while the increase in the liver index was significantly reversed in mice with TAX-Lips and PVP TAX-Lips treatment (6.4 ± 1.2% and 5.4 ± 0.9%) (6.4 ± 1.2% and 5.4 ± 0.9%) (*p* < 0.01; Table 2).

**TABLE 2:** Effect of TAX-Lips and PVP-TAX-Lips on the liver index in mice.

Group	Initial weight of mice(g)	Final weight of mice(g)	Liver weight(g)	Liver index (%)
Control	31.3 ± 3.0	35.5 ± 4.2	1.6 ± 0.3	4.4 ± 0.6
Model	30.5 ± 3.0	35.2 ± 5.0	2.6 ± 0.5^###^	7.5 ± 1.3^###^
TAX-Lips	31.8 ± 4.0	36.3 ± 5.4	2.3 ± 0.6^**^	6.4 ± 1.2^**^
PVP-TAX-Lips	32.2 ± 2.6	37.5 ± 4.1	2.0 ± 0.4^***^	5.4 ± 0.9^***^

As compared with the control group, ###*p* < 0.001; the model group, *p < 0.05, *p < 0.01, ***p < 0.001; data are presented as the mean ± SD (n = 8).

#### 3.5.3 PVP-TAX-Lips Attenuates Mice With ALI Induced by LPS/D-GalN

The levels of ALT, AST, GSH, MDA, and SOD were measured to evaluate the effect of PVP-TAX-Lips on LPS/D-GalN-induced ALI in mice. The levels of ALT and AST in the serum of mice were significantly increased in the model group after the intraperitoneal injection of LPS/D-GalN (*p <* 0.001), but the levels of ALT and AST were significantly reduced after TAX-Lips and PVP-TAX-Lips treatment (*p <* 0.01; Figures 4C,D). The levels of GSH and SOD were significantly reduced (*p <* 0.001) while the level of MDA was significantly increased (*p <* 0.01) in the liver tissue of the model group, while the abnormal expression levels of GSH, MDA, and SOD induced by LPS/D-GalN were significantly reversed by PVP-TAX-Lips treatment (*p <* 0.05; Figures 4E–G).

#### 3.5.4 Effect of PVP-TAX-Lips on Inflammatory Factors in Mice With ALI Induced by LPS/D-GalN

**FIGURE 5:**
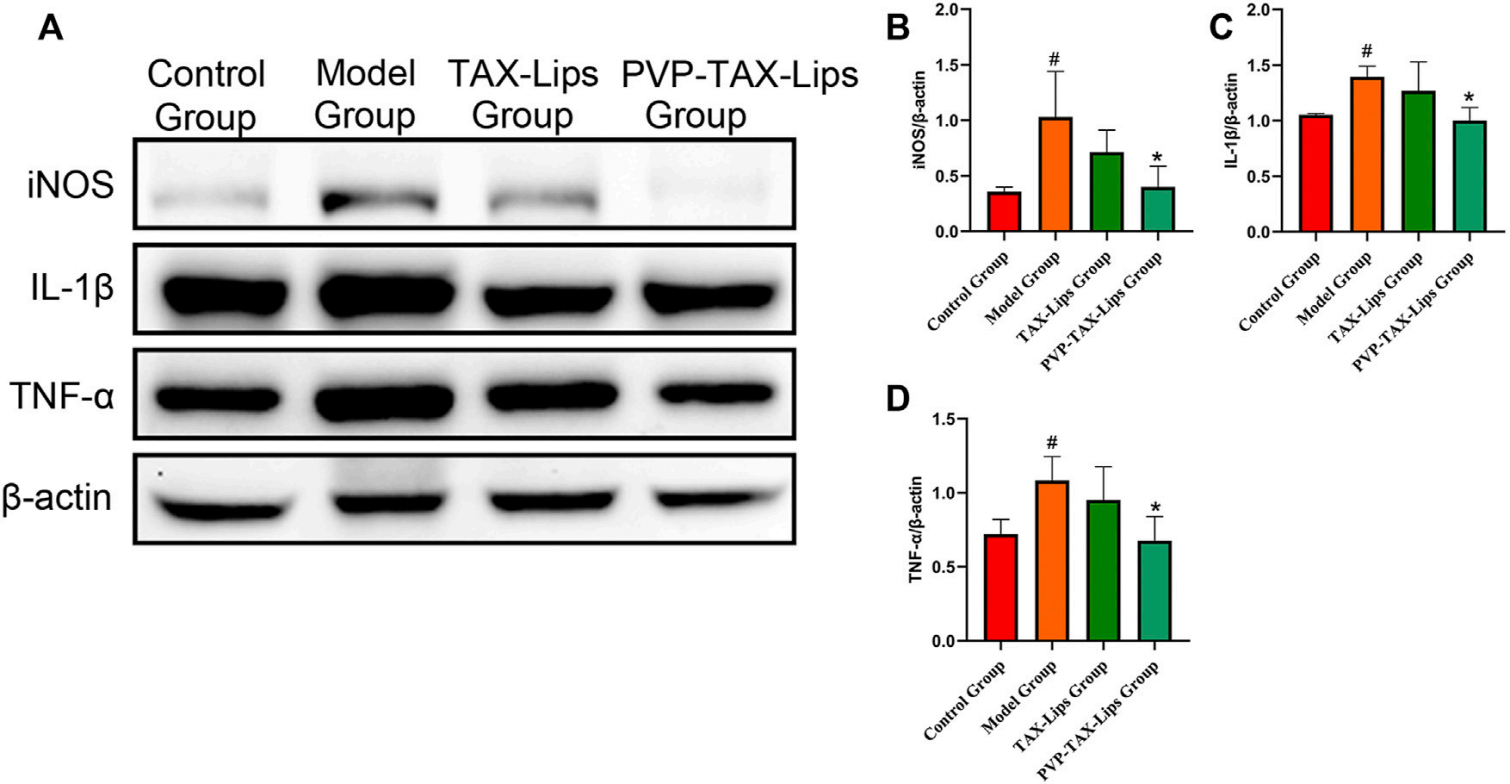
Inflammatory factors in mice with ALI induced by LPS/D-GalN. **(A)** Protein levels of iNOS, IL-1β, TNF-α, and β-actin analyzed by Western blotting. **(B–D)** Relative band intensity analyzed by ImageJ Analysis System, and β-actin was used as a control for equal loading. As compared with the control group, #*p* < 0.05. As compared with the model group, *p < 0.05. The data are presented as the mean ± SD (*n* = 3).

The expression levels of inflammatory factors iNOS, IL-1β, and TNF-α in LPS/D-GalN-induced ALI in mice were studied in order to estimate the anti-inflammatory effect of PVP-TAX- Lips (Figure 5). The protein expression levels of iNOS, IL-1β, and TNF-α were significantly increased in the model group compared with the control group after intraperitoneal injection of LPS/D-GalN (*p* < 0.05), whereas the protein expression levels were significantly decreased in the PVP-TAX-Lips treatment group compared with the model group (*p* < 0.05).

#### 3.5.5 PVP-TAX-Lips Improved LPS/D-GalN Induced ALI by Inhibiting the TLR4/NF-κB Signaling Pathway

**FIGURE 6:**
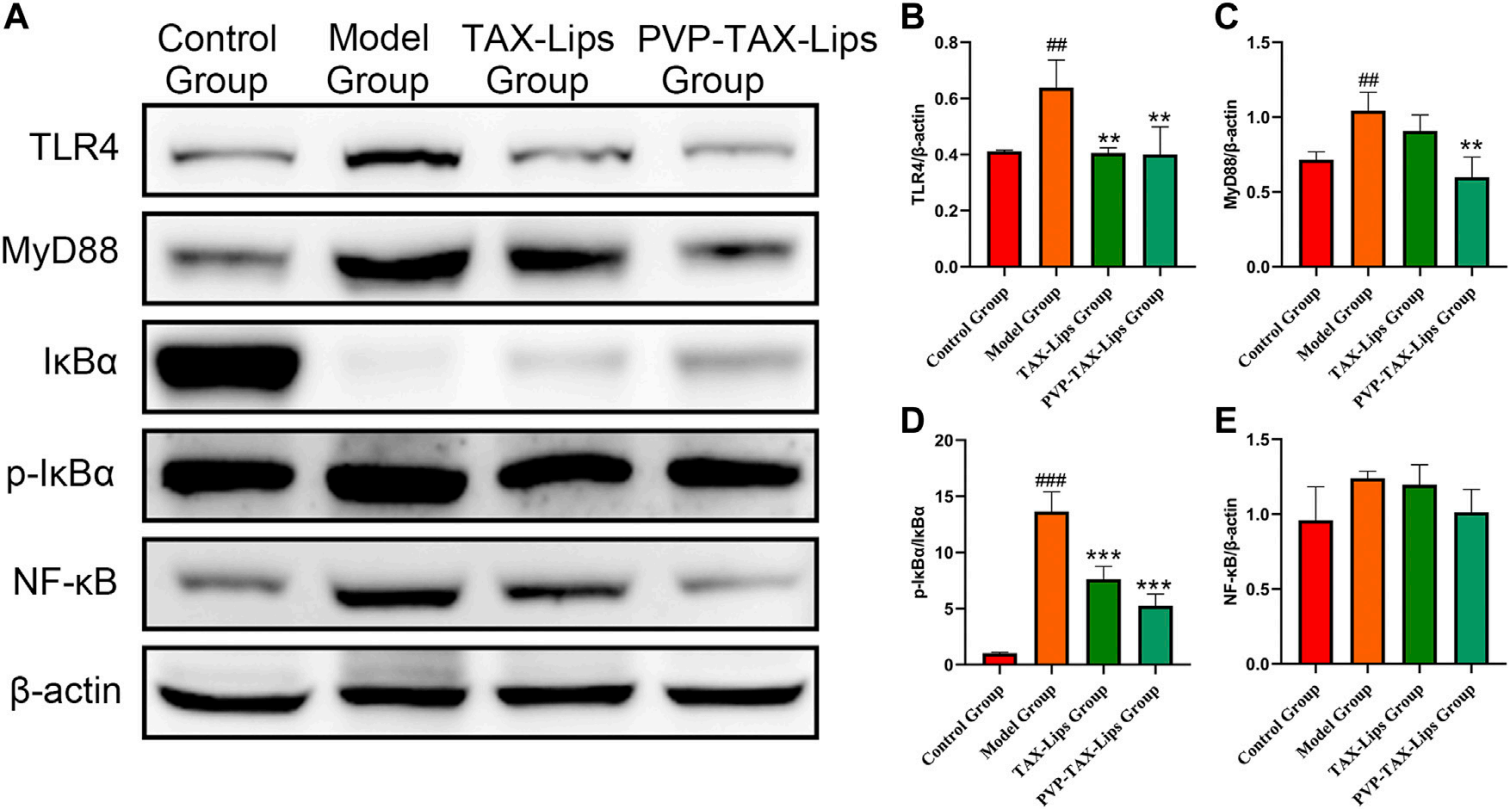
Expression of TLR4/NF-κB signaling pathway induced by LPS/D-GalN. **(A)** Protein levels of TLR4, MyD88, IκBα, p-IκBα, NF-κB, and β-actin analyzed by Western blotting. **(B–E)** Relative band intensity analyzed by ImageJ Analysis System, and β-actin was used as a control for equal loading. As compared with the control group, ##*p* < 0.01, ###*p* < 0.001; As compared with the model group, **p < 0.01, ***p < 0.001. The data are presented as the mean ± SD (*n* = 3).

The activation of TLR4/NF-κB signaling pathway plays an essential role in ALI induced by LPS/D-GalN. Therefore, the expression levels of related proteins TLR4, MyD88, IκBα, p-IκBα, and NF-κB were evaluated (Figure 6A), to study the effect of LPS/D-GalN-induced TLR4/NF-κB signaling pathway. The protein expression levels of TLR4, MyD88, p-IκBα/IκBα, and NF-κB were increased in the model group compared to the control group, while the protein expression levels were significantly inhibited after PVP-TAX-Lips treatment compared with the model group (Figures 6B–F). These results indicate that the hepatoprotective effect of PVP-TAX-Lips on LPS/D-GalN-induced ALI may be related to TLR4/NF-κB signaling pathway.

#### 3.5.6 PVP-TAX-Lips Improved LPS/D-GalN-Induced ALI by Increasing Autophagy

**FIGURE 7:**
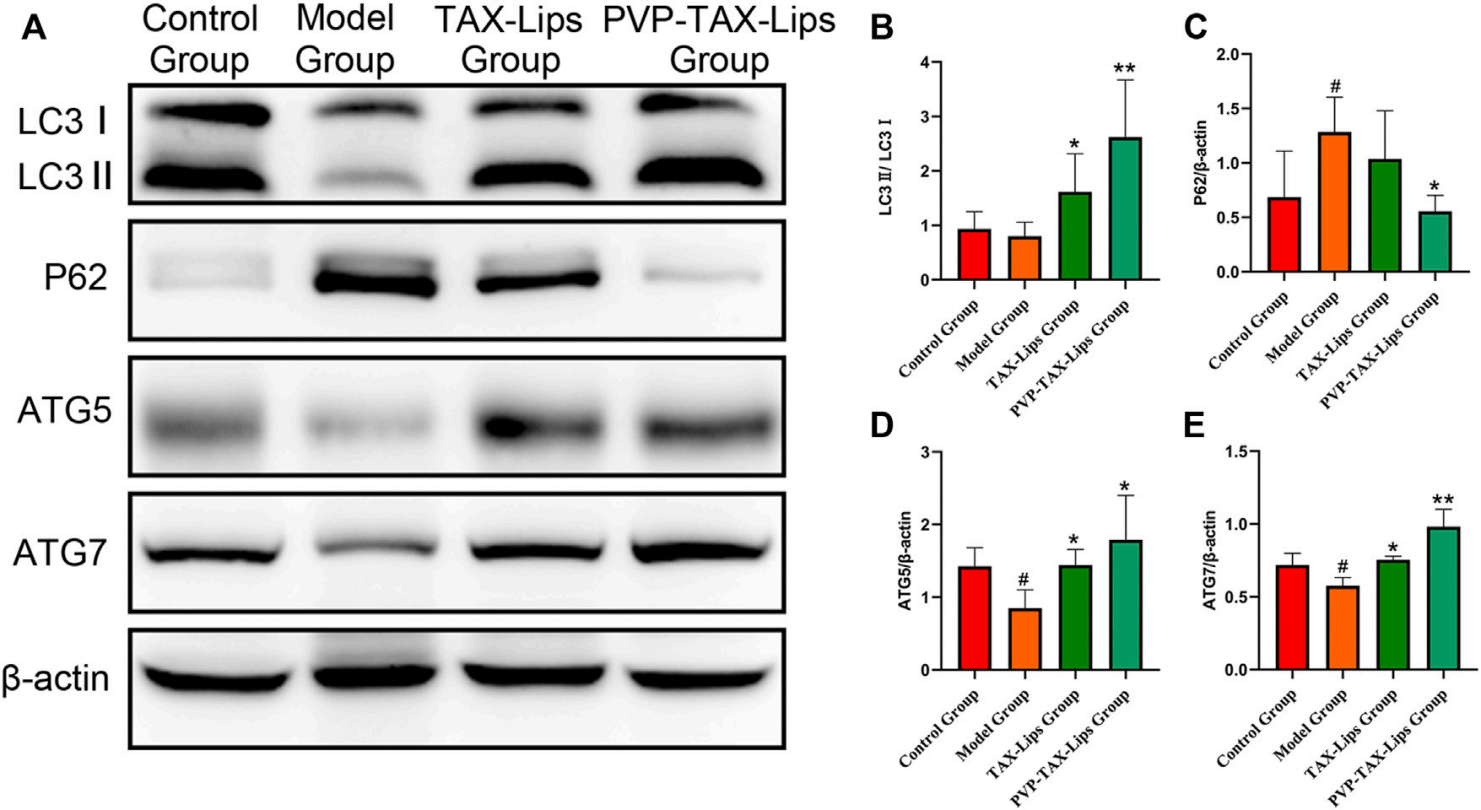
Expression of the autophagy proteins induced by LPS/D-GalN. **(A)** Protein levels of LC3, P62, ATG5, ATG7, and β-actin analyzed by Western blotting. **(B–E)** Relative band intensity analyzed by ImageJ Analysis System, and β-actin was used as a control for equal loading. As compared with the control group, #*p* < 0.05. As compared with the model group, *p < 0.05, **p < 0.01. The data are presented as the mean ± SD (*n* = 3).

Autophagy is involved in the cellular clearance of damaged organelles and pathogens, among other activities (Zhang et al., 2020). The protein expression levels of LC3II/I, ATG5, and ATG7 were reduced after the intraperitoneal injection of LPS/D-GalN in the model group mice, while the protein expression level of P62 was significantly increased (*p* < 0.05; Figure 7). In addition, PVP-TAX-Lips significantly reversed the effect of LPS/D-GalN on autophagy protein in mice (*p* < 0.05; Figure 7). In conclusion, the abovementioned results suggest that PVP-TAX-Lips avoided LPS/D-GalN-induced ALI by regulating the expression levels of autophagy protein.

## 4 Discussion

**FIGURE 8:**
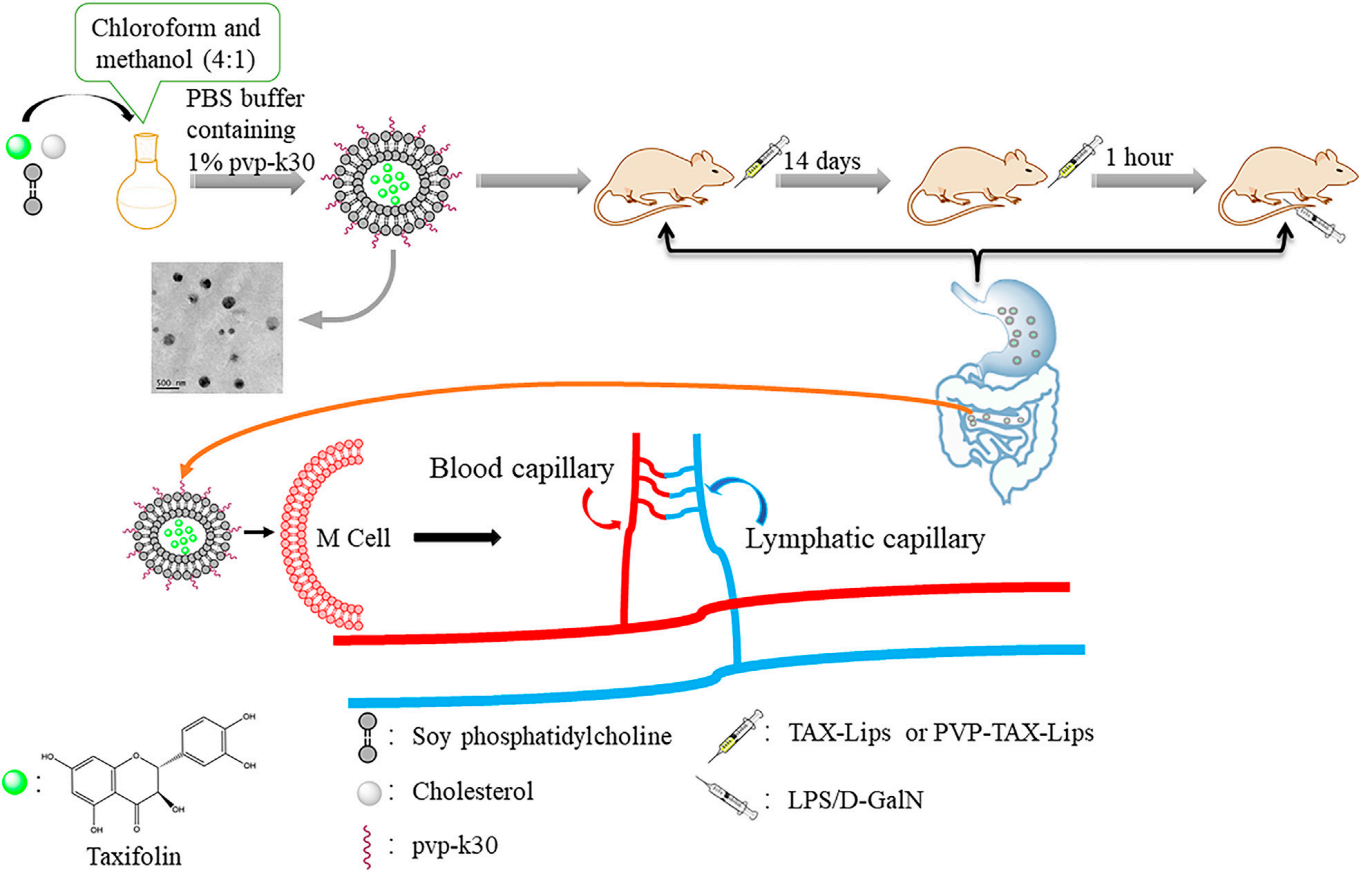
The diagram of PVP-TAX-Lips oral administration into the body circulation.

TAX is a flavonoid compound that has been proved to possess significant hepatoprotective effect in the previous studies (Hu et al., 2019; Sunil and Xu 2019; Yang et al., 2019). However, the poor water solubility of TAX limits its application, and for that it is important to find a method that improves the bioavailability of TAX, and liposomes are known to be a dosage form capable of enhancing the apparent water solubility of drugs. However, traditional liposomes have some disadvantages, as they are not suitable for oral administration due to the destruction of lipid membrane in the complex gastrointestinal environment, which leads to drug leakage in the gastrointestinal tract. Chemical modification of liposomes helps to reduce the effect of the complex gastrointestinal environment on the traditional liposomes, and intact liposomes can enter M cells through the intestinal epithelial cells to release drugs, thus prolonging their *in vivo* circulation (Figure 8). Therefore, it is crucial to find materials to modify traditional liposomes in order to improve the outcome of their oral administration. PVP is a drug carrier material with the advantages of good biocompatibility, low toxicity, and enhanced drug solubility (Edikresnha et al., 2019), which was employed here to modify TAX-Lips. PVP has been widely used in drug delivery systems, wound and burn dressings, and ophthalmic applications (Ajji et al., 2020; Teodorescu et al., 2019). According to the FTIR results, PVP-K30 was successfully located on the outer layer of TAX-Lips. The comparison of PVP-TAX-Lips with TAX-Lips revealed that the particle size of PVP-TAX-Lips was increased by 13.09 nm and the zeta potential of PVP-TAX-Lips was increased by 17.37 mV, which may improve the stability of the drug carrier system due to electrostatic repulsion (Hanafy and Ei-kemary 2022), thus reducing the aggregation between liposomes. The *in vitro* release results have demonstrated that PVP-TAX-Lips exhibited superior slow-release compared to free TAX and TAX-Lips, which, in turn, may prevent the leakage of TAX from liposomes in the simulated gastrointestinal fluid environment. This provided a basis for the hepatoprotective effect of PVP-TAX-Lips against LPS/D-GalN-induced ALI.

ALI has become one of the diseases that seriously damage human heath: the improper use of drugs, excessive alcohol consumption, and environmental pollution damages the hepatocytes, which induce serious consequences such as liver fibrosis, cirrhosis, and even liver cancer (Buchanan and Siclair 2021; Bunchorntavakul and Reddy 2018; Liu et al., 2021; Xiong and Guan 2017; Yang et al., 2019). Therefore, the development of new hepatoprotective drugs is a priority. The ALT and AST levels are important indicators to assess liver function, whereas GSH, MDA, and SOD are the biomarkers of the degree of oxidation *in vivo* (Bohm et al., 2016; Khoury et al., 2017; Zhou Honghong et al., 2018; Hanafy 2021; Huang et al., 2021). In this study, the expression levels of ALT, AST, GSH, MDA, and SOD were abnormally altered in the model group of mice after the intraperitoneal injection of LPS/D-GalN, while PVP-TAX-Lips were able to reverse this abnormal expression levels. Oxidative stress is one of the important factors for evaluating aging and diseases (Liu Aiyun et al., 2018; Yan et al., 2018), leading to inflammatory infiltration of neutrophils, structural destruction of hepatocytes, increased fat vacuolation, and irregular cell arrangement, as well as these changes can be observed by histopathological staining. The histopathological results presented here showed significant histopathological manifestations of liver injury compared with the control group, while PVP-TAX-Lips reduced the inflammatory infiltration of liver neutrophils and protected the liver structures with significant hepatoprotective effects.

LPS/D-GalN-induced ALI in mice not only produces oxidative stress but also inflammation (Liu et al., 2017a). When the hepatocytes are attacked by LPS, TLR4 recognizes LPS and activates nuclear transcriptional factor NF-κB with the help of MyD88. Hence, TLR4/NF-κB pathway is one of the important signaling pathways in LPS-D-GalN-induced ALI (Jia et al., 2018). The LPS/D-GalN activates TLR4/NF-κB signaling pathway and stimulates the release of proinflammatory factors, such as iNOS, IL-1β, and TNF-α. This study investigated the changes in expression levels of TLR4/NF-κB signaling pathway related factors and proinflammatory factors by PVP-TAX-Lips treatment of LPS/D-GalN-induced ALI, and the results of Western blot analysis showed that PVP-TAX-Lips significantly inhibited the activation of the TLR4/NF-κB signaling pathway and the expression levels of proinflammatory factors, confirming the involvement of this signaling pathway in the hepatoprotection mechanism of PVP-TAX-Lips.

Autophagy is a highly conserved mechanism of intracellular homeostasis that includes five different stages: initiation, extension, autophagosome formation, autophagosome lysosome fusion, and then degradation (Yang et al., 2010; Zhang et al., 2020). Autophagy can clearly damage cells and organs, so the increase of autophage protein expression helps to remove damaged cells and effectively prevent ALI induced by LPS/D-GalN in mice. Importantly, LC3, P62, ATG5, and ATG7 are the key nodes of the autophagy signaling pathway (Liu et al., 2019; Rah et al., 2017). The results of Western blot analysis were consistent with the fact that PVP-TAX-Lips treatment increases the levels of autophagy in mice and improves the ALI induced by LPS/D-GalN in mice, a result that is in line with a previous one, that showed that the upregulation of the autophagy signaling pathway effectively inhibited ALI (Zhang et al., 2020).

Current studies have shown that TLR4/NF-κB signaling pathway was closely related to autophagy (Lee et al., 2019). TLR4 is the main receptor of LPS and autophagy prevented LPS-induced injury and regulated the downstream role of TLR4 signaling pathway, which further confirmed the relationship between TLR4 signaling pathway and LPS-induced autophagy (Leventhal et al., 2016). It has been shown that LPS inhibits the expression levels of inflammatory factors such as TNF-α and IL-1β in macrophages through the activation of TLR4-MyD88 signaling pathway (Zhao et al., 2019), and rapamycin was found to attenuate brain injury and activate autophagy through the TLR4-MyD88 signaling pathway (Li et al., 2019). Recent studies also revealed the relationship between ALI and TLR4/NF-κB signaling pathway through LPS/D-GalN-induced ALI in mice, but the hepatoprotective mechanism of autophagy in combination with TLR4/NF-κB has not been yet explored (Chen et al., 2021; Jia et al., 2018).

## 5 Conclusion

In this research, PVP-TAX-Lips with hepatoprotective properties were prepared for the first time, expanding the therapeutic applications of TAX, a very poorly soluble compound from natural origin. Our work explored the hepatoprotective effect of PVP-TAX-Lips on the LPS/D-GalN-induced ALI in mice by regulating the expression levels of autophagy proteins and inhibiting the expression levels of TLR4/NF-κB signaling pathway with the activation of related inflammatory factors, which provided a new idea for TAX application and the prevention of ALI in the future. However, the preparation process of PVP-TAX-Lips remains to be optimized, and further studies on the hepatoprotection mechanism are needed to fully characterize the proposed drug delivery system.

## Data Availability

The original contributions presented in the study are included in the article/Supplementary Material. Further inquiries can be directed to the corresponding authors.
